# Reelin depletion alleviates multiple myeloma bone disease by promoting osteogenesis and inhibiting osteolysis

**DOI:** 10.1038/s41420-021-00608-8

**Published:** 2021-08-25

**Authors:** Aixia Dou, Ying Zhang, Yongjing Wang, Xiaoli Liu, Yanan Guo

**Affiliations:** Department of Hematology, The Second Hospital, Cheeloo College of Medicine, Shandong University, Jinan, Shandong China

**Keywords:** Cell signalling, Tumour biomarkers

## Abstract

Extracellular matrix glycoprotein Reelin is associated with tumor metastasis and prognosis in various malignancies. However, its effects on multiple myeloma (MM) are not fully understood. Here, we investigated the regulatory effects of Reelin on MM and its underlying pathogenic mechanisms. Lentivirus plasmid containing short hairpin RNA targeting Reelin (LV3-Reln) was transfected into SP2/0 cells to knockdown Reelin expression. Flow cytometry assay analyzed cell cycle and apoptosis while Transwell assay evaluated invasiveness. BALB/c mice were inoculated with LV3-Reln-transfected SP2/0 cells to establish MM model. Primary myeloma cells and osteoblasts/osteoclast were isolated from tumor tissue and limb long bones respectively. ELISA examined serum biomarkers and immunohistochemistry detected immunoglobulin light chain expression. Morphological changes and osteoclast/osteoblast differentiation were observed by histological staining. mRNA and proteins expression were determined by qPCR and WB. In vitro studies showed that Reelin depletion regulated osteolysis and osteogenesis balance, cell cycle, invasiveness, and apoptosis in SP2/0 cells. In LV3-Reln mice, tumor growth and invasiveness were suppressed, meanwhile, reduced osteoclast activation and enhanced osteoblast activity were observed. Reelin knockdown alleviated extramedullary morbidity and inhibited spleen immune cell apoptosis by down-regulating CDK5, IL-10, and Cyto-C expression. Furthermore, reduced Reelin expression restrained osteoclast differentiation while promoted osteogenesis in the bone of LV3-Reln mice. This was further supported by down-regulation of osteolytic specific mRNAs and proteins (Trap, Mmp9, Ctsk, Clcn7) and up-regulation of osteogenic specific ones (COL-1, Runx2, β-Catenin). Reelin exerted important impacts on myeloma development through rebalancing osteolysis and osteogenesis, thus might be a potential therapeutic target for MM.

## Background

Multiple myeloma (MM) is a neoplastic plasma-cell disorder characterized by hypercalcemia, anemia, renal insufficiency and bone disease, among which bony lesion developed in almost 80% of newly-diagnosed patients [1]. Accumulating genetic and epigenetic alterations of plasma cells contribute to the unbalanced ratio between osteoclast (OC) and osteoblast (OB) in favor of the first in multiple myeloma bone disease (MMBD) [2]. This imbalance centers around malignant transformation of MM cells and their aberrant interactions with bone marrow microenvironment, and ultimately leads to osteolytic bone lesions [3, 4]. In this complex process, osteoblastogenesis-specific factors, like Dickkopf-1 (DKK1) and Runt-related transcription factor 2 (Runx2), and osteoclastogenesis-specific ones, like nuclear factor kappa-B ligand (RANKL) and osteoprotegerin (OPG), are stimulated or inhibited by MM cells and establishes a positive feedback that sustains myeloma cell survival and subsequently causes continuous bone destruction [5]. Despite multidisciplinary approaches are used in MM treatment, including bisphosphonates, immunomodulators, and proteasome inhibitors, the median overall survival of MM remains short and bone lytic lesions hardly heal [4]. Further mechanistic explorations are required to give a profound understanding of MMBD, based on which patients could benefit from novel biomarkers and therapies.

Reelin, synthesized as a 3460-amino acid precursor protein with a molecular weight of 410 kDa, is a multifunctional extracellular glycoprotein with wide distribution in various tissues [6]. The most well-known function of Reelin involves regulating embryonic brain development and adult neurological disorders. To be more specific, Reelin controls neuronal radial migration, proper positioning, neuron maturation and synaptic formation at cellular level [7, 8]. On the other hand, recent studies found Reelin expression was altered in a variety of cancers. In esophageal carcinoma, high Gleason score prostate cancer, retinoblastoma and non-Hodgkin lymphoma, Reelin expression was highly induced [9–13]. Moreover, growing evidence implies this upregulation of Reelin is imperative for tumor metastatic progression [12, 14, 15]. In contrast, the down-regulation trend of Reelin was observed in hepatocellular carcinoma, breast, gastric, and pancreatic cancers [16–19]. Interestingly, it is reported that high Reelin expression correlates with poor prognosis in MM patients [20]. Through targeting integrin/focal adhesion kinase (FAK), Reelin promotes adhesion of MM cells to bone marrow stromal cells [21, 22]. Also, Reelin plays a critical role in facilitating MM cell proliferation and glycolysis by activating FAK/Syk/Akt/mTOR and STAT3 pathways [20]. However, as a frequently happened complication of MM, whether Reelin exert impacts on MMBD and influence OB/OC balance is still unclear, hindering its exploitation as a potential therapeutic target.

In the present study, we investigated Reelin’s role in the regulation of MMBD. We further explored whether Reelin depletion alters myeloma cell proliferation, apoptosis, and invasiveness, thus influence OC and OB activity, and rebalance osteolysis and osteogenesis.

## Results

### Reelin depletion regulated osteolysis and osteogenesis balance, cell cycle, apoptosis, and invasiveness in vitro

**Fig. 1:**
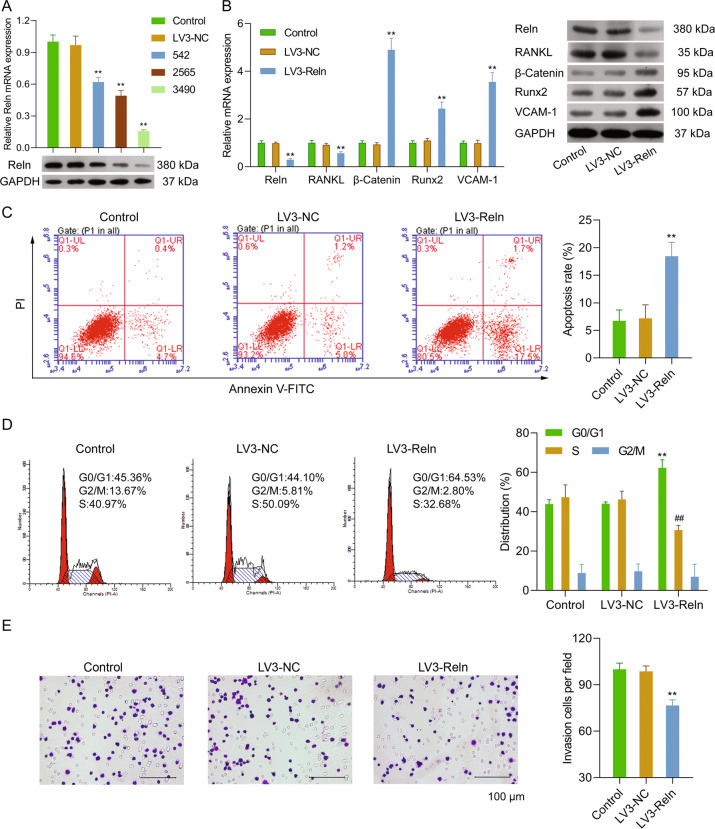
Reelin depletion regulated osteolysis and osteogenesis balance, cell cycle, apoptosis, and invasiveness in vitro. **A** Three lentivirus plasmids containing short hairpin RNA (shRNA) for Reelin (LV3-Reln-542/2565/3490) or the corresponding negative control (LV3-NC) were transfected into SP2/0 cells. LV3-Reln-3490 was chosen to complete this study as Reelin expression was best inhibited in this group. **B** mRNA and protein expression of Reelin and osteoclast-/osteoblast-related genes detected by qRT-PCR and western blotting respectively. **C** Apoptosis of control, LV3-NC and LV3-Reln SP2/0 cells detected by Annexin V/PI double staining. **D** Cell cycle analysis of SP2/0 cells infected with LV3-NC and LV3-Reln by flow cytometry. **E** Transwell assay showed that invasiveness in LV3-Reln group were inhibited. Reln stands for Reelin. ^**^p < 0.01, ^##^*p* < 0.01 vs. Control group.

To examine the effects of Reelin on MM in vitro, we knocked down Reelin expression by transfecting lentivirus plasmid containing short hairpin RNA targeting Reelin (LV3-Reln) into SP2/0 cells (Fig. 1A). Cells transfected with blank plasmid was used as negative controls (LV3-NC). LV3-Reln-3490 was chosen to complete this study as Reelin expression was best inhibited in this group. RT-PCR and western blotting were applied to detect key factors involved in lytic lesion development. The mRNA and protein expression of pro-osteoclastogenic factor, RANKL, was significantly decreased while those of pro-osteoblastogenic factors, β-Catenin and Runx2 were increased in LV3-Reln cells in comparison to LV3-NC ones, indicating a regulation favored osteogenesis (Fig. 1B). VCAM-1 is a notable surface protein that participated in intercellular adhesion. Here, we determined that in SP2/0 cells, VCAM-1 mRNA and protein expression were elevated by Reelin knockdown, which implied improved adhesion ability (Fig. 1B). We then performed flow cytometry and observed that Reelin depletion significantly enhanced cell apoptosis (apoptosis rate: *p* < 0.05 vs. control, Fig. 1C). Also, we investigated whether Reelin impacts cell cycle. Decrease in S phase and increase in G0/G1 phase were found in LV3-Reln SP2/0 cells, suggesting Reelin downregulation induced cell cycle progression from G1 to S phase (Fig. 1D). Furthermore, SP2/0 cell invasiveness was attenuated in LV3-Reln group (Fig. 1E).

### Inhibition of reelin suppressed tumor growth and invasiveness in vivo

**Fig. 2:**
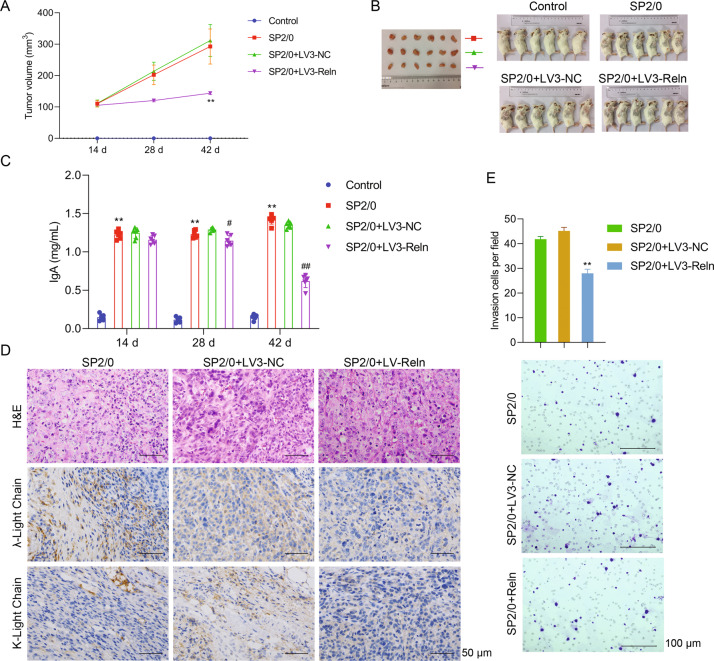
Inhibition of Reelin suppressed tumor growth and invasiveness in vivo. Eight-week-old female BALB/c mice injected with LV3-Reln- or LV3-NC-transfected SP2/0 cells to knockdown Reelin expression or served as negative control (*n* = 6 per group). **A** Tumor volume for every animal in all groups were detected at 14, 28, and 42 days. ^**^p < 0.01 vs. SP2/0 group. **B** Images of tumor tissue collected from corresponding animal groups after MM model was successfully established. **C** Serum IgA levels at 14, 28, and 42 days for each group were detected by ELISA. ^**^p < 0.01 vs. Control group. ^#^*p* < 0.05, ^##^*p* < 0.01 vs. SP2/0 group. **D** Representative H&E staining (upper one third panel) and immunoglobulin λ-/κ-light chain immunohistochemistry (lower two third panel) images in tumor tissues. **E** Transwell assay showed that invasiveness in LV3-Reln group were inhibited. Reln stands for Reelin. ^**^p < 0.01 vs. SP2/0 group.

After examined its regulatory roles in vitro, we explored whether Reelin can exert impacts on tumor development in vivo. For this purpose, we generated MM model by subcutaneously inoculating eight-week-old female BALB/c mice with SP2/0 cells. Mice injected with LV3-Reln- or LV3-NC-transfected SP2/0 cells to knockdown Reelin expression or served as negative control. As shown in Fig. 2A, B, compared to SP2/0 mice, tumor growth was significantly suppressed in LV3-Reln group during day 14–42 (*p* < 0.001 vs. SP2/0 group). At day 42, the mean tumor volume of LV3-Reln group was almost half of LV3-NC ones (LV3-Reln 143.9 mm^3^ ± 11.1 mm^3^ vs. LV3-NC 311.9 mm^3^ ± 124.4 mm^3^, *p* < 0.01). Since myeloma cells are neoplastic plasma cells which make monoclonal immunoglobulins, we detected the serum IgA levels at day 14, 28, and 42 to explore whether Reelin can inhibit the production of this monoclonal protein, thus suppressing the pathologic effects of MM. We found that LV3-Reln mice had significantly decreased IgA level since day 28 (*p* < 0.001 vs. SP2/0 group, *p* < 0.001 vs. LV3-NC group, Fig. 2C). In agreement with tumor volume and IgA level changes, fewer plasma cell infiltration and λ-/κ-light chain accumulation was found in LV3-Reln group, as visualized by H&E staining and immunohistochemistry examination (Fig. 2D). Then, we isolated and cultured primary MM cells from tumor tissues of each group. Using Transwell assay, we found the number of invasion cells was 28.0 ± 4.1/field in LV3-Reln group, significantly lower than SP2/0 group (45.2 ± 3.5/field, *p* < 0.001, Fig. 2E). Our data suggested that inhibiting Reelin expression in myeloma cells could suppress tumor growth and also restrain its invasive ability.

### Inhibition of reelin rebalanced osteoclasts and osteoblasts activity

**Fig. 3:**
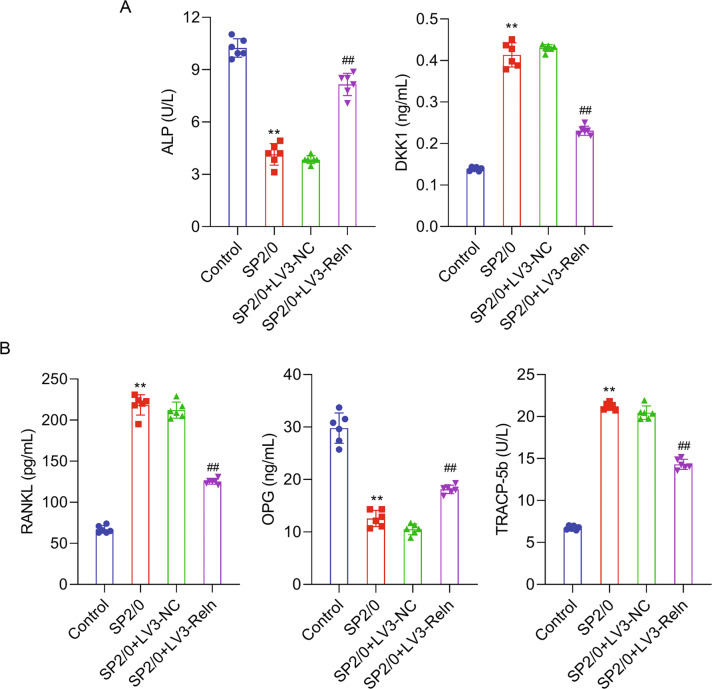
Inhibition of Reelin rebalanced osteoclasts and osteoblasts activity. **A** Serum osteoblast-related factors (ALP and DKK1) quantified by ELISA. **B** Serum osteoclast-related factors (RANKL, OPG, and TRACP-5b) quantified by ELISA. Reln stands for Reelin. ^**^p < 0.01 vs. Control group; ^##^*p* < 0.05 vs. SP2/0 group.

Bone disease characterized by osteolysis and osteopenia was a defining clinical manifestation of MM [23]. After observing Reelin’s role in suppressing MM tumor growth, we investigated the changes of key proteins involved in bone lesions. Relative to SP2/0 mice, the serum level of ALP was increased by Reelin knockdown (Fig. 3A), representing enhanced mineralization inside the bone tissue [24]. Meanwhile, the concentration of DKK1, which was secreted by osteoblasts and MM cells, was reduced by Reelin depletion, indication inhibited osteoblast maturation and thus less bone formation (Fig. 3A). On the other hand, serum OPG level was increased while RANKL level was decreased in LV3-Reln mice in comparison to SP2/0 ones (Fig. 3B). Since system RANKL/OPG ratio plays a vital role in activating osteoclast [1], our results showed this ratio was lowered by Reelin knockdown and thus inhibited osteoclast activity. Also, the level of bone resorption marker, TRACP-5b, was reduced in LV3-Reln group [25], further supporting increased osteolysis observed in LV3-Reln mice (Fig. 3B). Therefore, a delicate balance among osteoclast, osteoblast, and myeloma cells was regulated and maintained by Reelin depletion.

### Reelin knockdown attenuated extramedullary MM

**Fig. 4:**
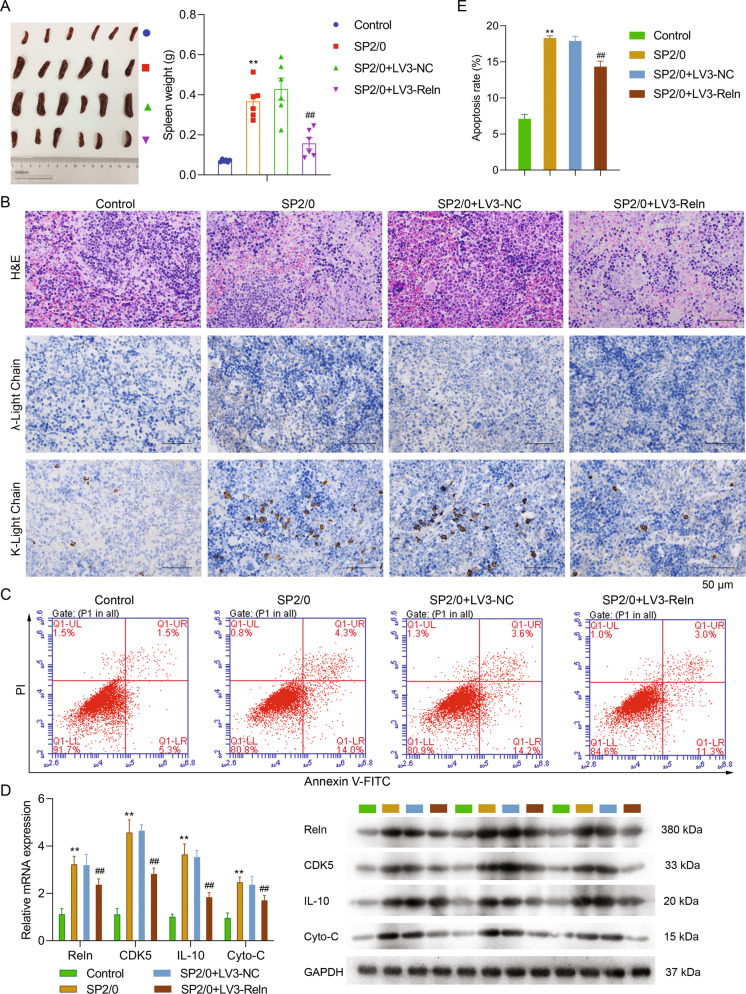
Reelin knockdown attenuated MM in spleen. **A** Spleen weight detected in control, SP2/0, LV3-NC and LV3-Reln groups (*n* = 6/group). **B** Representative H&E staining (upper one third panel) and immunoglobulin λ-/κ-light chain immunohistochemistry (lower two third panel) images in spleen tissues. **C**, **E** Apoptosis of immune cells in spleen tissue detected by Annexin V/PI double staining. And apoptosis rate was calculated for each group in (**E**). **D** mRNA and protein expression of apoptosis-related genes examined by RT-PCR and western blotting respectively and representative bands were shown in the right panel. Reln stands for Reelin. ^**^p < 0.01 vs. Control group; ^##^*p* < 0.01 vs. SP2/0 group.

Extramedullary MM is an aggressive part of MM with a subclone of plasma cells thriving outside bone marrow. To better understand this, we investigated the effects of Reelin on one of the most important peripheral immune organs, spleen. The mRNA and protein expression levels of Reelin were down-regulated in local spleen tissues of LV3-Reln mice. As displayed in Fig. 4A, spleen weight of these Reelin depletion mice were significantly lighter than SP2/0 ones (LV3-Reln 0.16 g ± 0.10 g vs. SP2/0 0.30 g ± 0.14 g, *p* < 0.001). Less plasma cells infiltration in the spleen of LV3-Reln mice was observed by H&E staining. Likewise, Fig. 4B shows that less λ- and κ-light chain expression was detected by immunohistochemistry staining. Importantly, in contrast with the alterations occurred in MM tissue, the apoptosis of immune cells within spleen was inhibited by Reelin down-regulation, suggesting greater functional immune cells participated in immune response (apoptosis rate: LV3-Reln 14.33% ± 0.75% vs. LV3-NC 17.9% ± 0.66%, *p* < 0.01, Fig. 4C, E). Regarding the regulatory mechanism underlying this phenomenon, we revealed that the expression of CDK5, IL-10, and Cyto-C were decreased in LV3-Reln group both at mRNA and protein level (Fig. 4D). Hence, our data supported that Reelin depletion attenuated spleen lesion in MM by inhibiting immune cell apoptosis, thus might maintain effective immune responses.

### Reduced reelin expression inhibited osteoclast differentiation and osteolysis

**Fig. 5:**
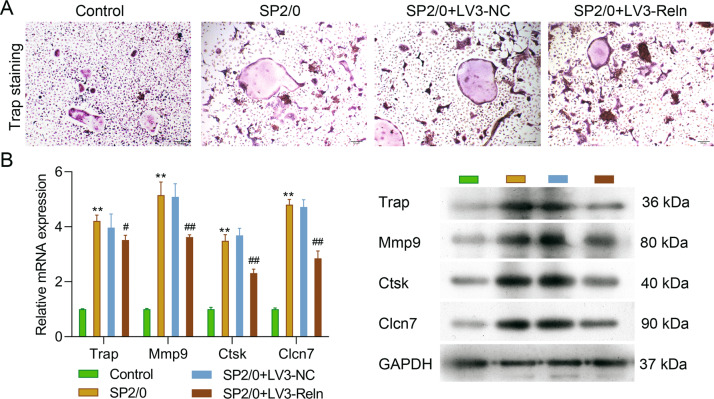
Reduced Reelin expression inhibited osteoclast differentiation and osteolysis. We investigated whether Reelin down-regulation can attenuate the generalized osteoclast activation in isolated osteoclasts (*n* = 6 per group). **A** Representative Trap staining images showing osteoclast activity for control, SP2/0, LV3-NC, and LV3-Reln groups. **B** mRNA and protein expression of osteoclastogenic genes examined by RT-PCR and western blotting respectively. Reln stands for Reelin. **p < 0.01 vs. Control group; ^#^*p* < 0.05, ^##^*p* < 0.01 vs. SP2/0 group.

It is well known that osteocyte-OC-OB axis was disrupted in MMBD, leading to clinical osteolytic manifestations. In order to investigate whether Reelin down-regulation can attenuate the generalized OC activation, we isolated and cultured OC from limb bones. As demonstrated in Fig. 5A with Trap staining, it is evident that the size of OC isolated from LV3-Reln mice were smaller than those from LV3-NC ones. Lytic enzymes that are biomarkers of osteoclast activity, such as TRAP, MMP9, and CTSK were down-regulated in LV3-Reln mice at mRNA and protein levels (Fig. 5B). Meanwhile, chloride ion channel 7 (CLCN7) mRNA and protein expressions were also restrained (Fig. 5B, C). Our results evidenced reduced Reelin expression inhibited osteoclast differentiation and osteolysis.

### Reelin knockdown promoted osteoblast differentiation and osteogenesis

**Fig. 6:**
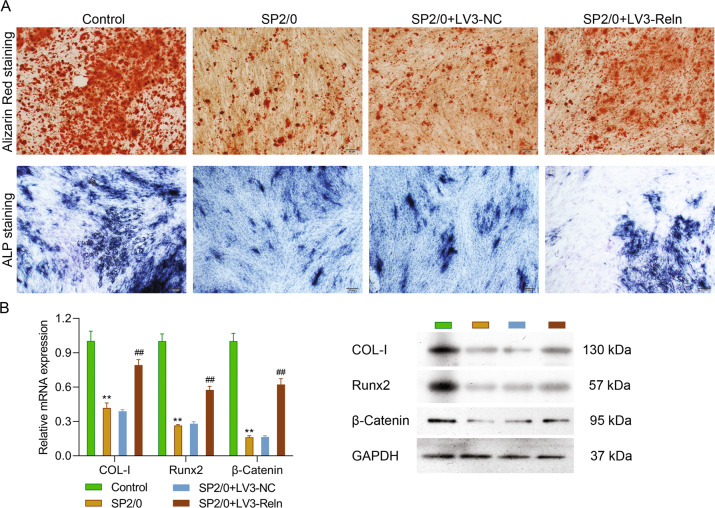
Reelin knockdown promoted osteoblast differentiation and osteogenesis. We isolated osteoblasts from limb bones and explored Reelin’s impacts on bone formation (*n* = 6 per group). **A** Representative Alizarin Red staining (upper panel) and ALP staining (lower panel) images showing osteoblast activity. **B** mRNA and protein expression of osteoblastogenic genes examined by RT-PCR and western blotting respectively. Reln stands for Reelin. ^**^p < 0.01 vs. Control group; ^##^*p* < 0.01 vs. SP2/0 group.

Besides osteolytic lesions, MMBD also displays osteogenesis dysfunction. Therefore, we isolated and cultured OB from limb bones and explored Reelin’s impacts on bone formation. As visualized by Alizarin Red staining in upper panel of Fig. 6A, the differentiation of stem cells towards OB was highly induced in LV3-Reln group relative to SP2/0 one. Similarly, ALP staining presented in the lower panel supported this finding. The Runx2 and Wnt/β-catenin pathways are key players in osteoblastogenesis. The expression of Runx2 and β-catenin mRNA and proteins are elevated 2–3 times by Reelin knockdown (Fig. 6B), representing improved OB activity. It is known that the activation of Runx2 induce osteogenic gene expression [26]. This is in accordance with what we observed here as COL-1 gene and protein production were increased in LV3-Reln group (Fig. 6B).

## Discussion

MM accounts for 1% of all tumors and represents 13% of hematologic cancers [3]. MMBD, characterized by osteoclast-mediated destructive bony lesions, is a devastating complication of MM and leads to hypercalcemia, osteoporosis, bone pain and fractures [1]. More importantly, myeloma cells directly and indirectly exert effects on OC and OB, thus contributing to tumor growth, survival and drug resistance [2]. Extracellular matrix protein Reelin was reported to participate in tumor cell progression, migration and adhesion, particularly in MM according to recent studies [20, 22]. However, whether Reelin affect MMBD in MM development is still unclear. In the present study, we found Reelin depletion reduced tumor progression in MM mice. Altered cell cycle, enhanced apoptosis and impaired invasiveness of myeloma cells were observed both in vitro and in vivo. Furthermore, consistent with its tumor attenuation function, Reelin knockdown alleviated MMBD by regulating OC and OB activity and rebalancing osteolysis and osteogenesis in MM model mice.

**Fig. 7:**
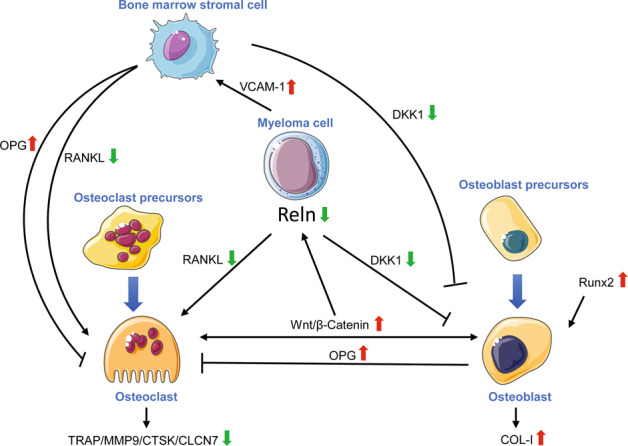
Schematic diagram showing the intercellular connection in LV3-Reln mice. The complex interaction between MM cells, bone marrow stromal cells, OC and OB are a dynamic process in MMBD, among which RANK/RANKL/OPG system plays pivotal roles. Reelin knockdown inhibited this important axis. Moreover, Reelin downregulation also regulated Runx2/ DKK1 and Wnt/β-catenin signaling, thus impacted the osteogenic function of OB.

The cardinal participant of bone remodeling is OC. The close interaction between bone marrow stromal cell, MM cell and extracellular matrix proteins are essential for osteoclastogenesis. This process was mediated by cytokines and growth factors secreted by these cells through autocrine or paracrine loops and altered the bone marrow microenvironment, among which RANKL is the most notable one [27]. Here, we found RANKL expression was decreased while OPG, a decoy receptor for RANKL, was increased in MM mice. These data implicated restrained RANK/RANKL/OPG signaling pathway activation, thus possibly diminished OC activity in MM (Fig. 7). In addition, in respond to RANKL expression, the transcription factor, nuclear factor of activated T cell-c1 (NFATc1), was activated and subsequently genes typified the OC lineage were induced, such as TRAP, MMP9, CTSK and CLCN7 [28]. These are proteinases secreted by mature osteoclasts in order to dissolve the inorganic and organic components of bone. The downregulation of them by Reelin knockdown just as observed in our investigation represented lowered activity of OC. Meanwhile, CLCN7 was proved to shunt lysosomal acidification and proton-pump currents [29], thus inhibition of CLCN7 in LV3-Reln MM mice can reduce dissolution of the inorganic bone matrix.

On the other hand, the MMBD alleviation also required to rectify the imbalanced OC/OB activity and promote bone synthesis. As visualized in Fig. 6A by Alizarin Red and ALP staining, new bone formation and OB maturation in LV3-Reln cells was promoted. Several molecular pathways were involved in this process. Runx2 is essential for osteoblast differentiation and chondrocyte maturation as upregulated expression was detected in pre-osteoblast and reaching the maximal expression level in immature OB [30]. According to MMBD patients bone marrow biopsies, the numbers of Runx2-positive OB and stromal cells in those with osteolytic lesions were greater than those without [5, 31]. Our data showed that Runx2 was downregulated by Reelin depletion in primary OB, suggesting enhanced OB differentiation towards mature cells in the stimulation of highly active bone resorption. Another critical pathway involved in OB differentiation is canonical Wnt pathway, and its effective transduction relies on stabilization of β-catenin while DKK1 was evidenced to antagonize Wnt pathway [5, 32]. As previously reported, DKK1 is expressed by MM cell and bone marrow stromal cells [1, 4]. Therefore, when MM developed, Reelin knockdown in MM cells restrained DKK1 expression and stimulated Wnt/β-catenin signaling pathway, leading to bone formation (Fig. 7).

Actually, the complex interaction between MM cells, bone marrow stromal cells, OC and OB are a dynamic and constant process in MMBD development (Fig. 7). To sustain this network, RANK/RANKL/OPG system among these cells plays the most important role but Reelin knockdown inhibited this pivotal axis. Moreover, Reelin downregulation also regulated Wnt/β-catenin signaling activation thus influenced its facilitation of MM cell migration, invasion, and adhesion [33, 34]. Wnt/β-catenin signaling is also involved in matching bone resorption with equal bone formation for that its secretion by OB finally activates signaling cascade to stimulate OC differentiation. Through intervening this crosstalk, Reelin knockdown helped to break this vicious cycle and alleviated MMBD. Through our observation, VCAM-1 expression was lowered by Reelin depletion, possibly reducing MM cell homing and suspending its proliferation [5]. OPG is not only expressed by OB, but also by bone marrow stromal cells [5], hence, our data found reduced Reelin expression blocked this synergy.

Inconclusion, our results suggested that Reelin exerted important impacts on myeloma development through rebalancing osteolysis and osteogenesis, thus might be a potential therapeutic target for MM.

## Methods

### Myeloma cell line culture and knockdown of Reelin in SP2/0 cells

The mouse multiple lymphoma cell line SP2/0 cell was purchased from ATCC (CRL8006). The cells were confirmed not contaminated by mycoplasma. SP2/0 cells were cultured in RPMI-1640 Medium (DMEM) supplemented with 10% fetal bovine serum (FBS) and 1% penicillin-streptomycin in a 5% CO_2_/water-saturated incubator at 37 °C. Three lentivirus plasmids containing short hairpin RNA (shRNA) for Reelin (LV3-Reln-542/2565/3490) or the negative control (LV3-NC) were infected into SP2/0 cells. Lentivirus used to knock down Reelin was designed by GenePharma (Shanghai) and the targeted sequences were listed in Table 1. Stably infected SP2/0 cells were selected and used for establishing animal model.

**Table 1:** Targeted sequences of Reelin by lentivirus.

Name	Sequence (5ʹ to 3ʹ)
LV3-NC	TTCTCCGAACGTGTCACGT
LV3-Reln-Mus-542	GCACCTTCTTTGATGGCTTGC
LV3-Reln-Mus-2565	GCAGCTAATCACGTCCTTTCT
LV3-Reln-Mus-3490	GCACTTCCTTCCACGATTATG

### MM mice model establishment

Six-week-old BALB/c female mice were purchased from Laboratory Animal Center, Sun Yat-Sen University. To establish MM mice model, we subcutaneously injected mice with 100 μL LV3-NC- or LV3-Reln-transfected SP2/0 cell suspense (2 × 10^6^ cells) on the chest. The control group was injected with PBS. Tumor size was measured every 2 weeks and the survival state of each mice were observed. All mice were nurtured under controlled conditions (12-hour/12-hour light/dark cycle). After adapted to feeding and environment, mice were randomly assigned to each study group (at lear 6 animals per group). No blinding was applied and no statistical methods were used to estimate sample size. All animal experiments were approved by the Animal Care and Use Committee of The Second Hospital, Cheeloo College of Medicine, Shandong University, and humane care was provided according to the criteria stated in the *Guide for the Care and Use of Laboratory Animals* prepared by the National Academy of Sciences and published by the National Institutes of Health.

### Mice primary cells isolation and cell culture

Primary OB and OC were isolated from limb long bones of each group as previously described [35]. OC cells were co-cultured with bone flaps for 60 min and then induced in α-MEM medium supplemented with 15% fetal bovine serum (FBS) and 1% penicillin-streptomycin. OB Cells were cultured in F-12 medium supplemented with 10% fetal bovine serum (FBS) and 1% penicillin-streptomycin. Both cells were incubated in a 5% CO_2_/water-saturated incubator at 37 °C.

### RNA extraction and qRT-PCR

Total RNA was extracted from SP2/0 cells, spleen specimens or cultured osteoclasts/osteoblasts using TRIzol (Takara Bio, Tokyo, Japan) according to the manufacturer’s instructions, followed by complementary DNA synthesis. Quantitative real-time PCR was performed with the Bestar® SYBR Green PCR MasterMix (DBI, Shanghai, China). The specific primers for each gene were listed below (Table 2). The targeted gene transcription levels were calculated by normalizing to β-actin expression. Each experiment was repeated for at least three times.

**Table 2:** Primers used in qRT-PCR analyses.

Gene	Forward primer (5ʹ-3ʹ)	Reverse primer (5ʹ-3ʹ)
β-actin	CATTGCTGACAGGATGCAGA	CTGCTGGAAGGTGGACAGTGA
Reelin	TGCTGCAGTACAGTGTCAAC	GACCTCCACATGGTCCAAAG
RANKL	TTTGCACACCTCACCATCAA	ATGCCGAAAGCAAATGTTGG
RUNX2	TTCAACGATCTGAGATTTGTGGG	GGATGAGGAATGCGCCCTA
β-Catenin	TGCGCATGGAGGAGATAGTA	TGGAGTAACTCTGTCAGGGG
VCAM-1	GCCTCAACGGTACTTTGGAT	ATGGAGTCACCGATTTGAGC
CDK5	CCCTGAGATTGTGAAGTCATTCC	CCAATTTCAACTCCCCATTCCT
IL-10	TTGGGTTGCCAAGCCTTATC	TGATTTCTGGGCCATGCTTC
Cyto-C	CCAAATCTCCACGGTCTGTTC	ATCAGGGTATCCTCTCCCCAG
MMP9	CTGGACAGCCAGACACTAAAG	CTCGCGGCAAGTCTTCAGAG
Trap	CACTCCCACCCTGAGATTTGT	CATCGTCTGCACGGTTCTG
Ctsk	GAAGAAGACTCACCAGAAGCAG	TCCAGGTTATGGGCAGAGATT
Clcn7	CGCCAGTCTCATTCTGCACT	GCTTCTCGTTGTGTGGAATCT
COL-1	GCTCCTCTTAGGGGCCACT	CCACGTCTCACCATTGGGG

### Western blotting

Total protein was extracted from mouse spleen tissue samples or cultured cells in lysis buffer and quantified using the BSA method (FA016-50G, Amresco). Each protein sample (20 μg) was subjected to sodium dodecyl sulfate (SDS)/polyacrylamide gel electrophoresis (PAGE) and transferred to a polyvinylidene fluoride (PVDF) membrane (IPVH00010, Millipore) that was incubated with primary antibody at 4 °C overnight and secondary antibody (HRP Goat anti-Rabbit IgG, #BA1054, 1:20,000; Wuhan Boster Biological Technology, Ltd., Wuhan, China; HRP Goat anti-Mouse IgG #BA1051, 1:20,000; Wuhan Boster Biological Technology) at ambient temperature for 40 min. Densitometric analysis was performed on the autoradiogram (Bio-5000, MICROTEK). The protein expression levels were quantified and normalized to GAPDH. Primary antibodies used in this study: Reelin, 1:2000, #ab78540; β-catenin, 1:4000, #ab16051; RANK, 1:1000, #ab45039; Runx2, 1:1000, #ab23981; VCAM-1, 1:5000, #ab134047; CDK5, 1:2000, ab172730; IL-10, 1:1000, ab189392; Cyto-C, 1:5000, ab133504; Trap, 1:10000, ab133238; MMP9, 1:1000, ab228402; Ctsk, 1:1000, ab187647; Clcn7, 1:500, ab136016; COL-1, 1:1000, ab255601; GAPDH, 1:10000, #ab8245. Each experiment was repeated for at least three times and representative bands were shown.

### Trap/Alizarin red/ALP staining

Trap staining: isolated primary OC were washed twice with cold PBS after removing medium. To confirm the morphology of OC, cells were lysed with 0.05% Triton-X100 at 4 °C, fixed with 3.7% formalin, and then permeablized with 0.1% Triton X-100 and finally stained for TRAP with the leukocyte acid phosphatase kit (Sigma). The images of TRAP-positive cells were captured under a light microscope. Alizarin Red and ALP staining: isolated OB cells from each group were stained with Alizarin Red (Solarbio, Beijing, China) and ALP (Jiancheng, Nanjing, China) according to manufacturer’s instruction to visualize their morphology.

### HE staining and immunohistochemistry

To visualize the histological changes, hematoxylin and eosin (H&E) staidning were performed on paraffin-embedded tumor and spleen sections that were cut into 4 μm slices. Immunohistochemistry staining were performed on paraffin-embeded tumor and liver specimens that were cut into 4 μm slices. The expression and localization of λ-/κ-light chain of immunoglobulin were investigated with a as primary antibody (λ- light chain, #bs-3801R, Bioss, Beijing, China; κ-light chain, ab190484/ bs-0330R, Bioss, Beijing, China) and were subsequently treated with secondary antibody of corresponding species. Images of the histological staining were acquired with a light microscope.

### Transwell assay

To measure the invasiveness of MM cells, 8-μm pore membrane filter chamber kit was used (Costar 3422, Corning, Inc.). The isolated cells were first infected with plasmids for 2 days. Then they were resuspended with serum-free medium while the bottoms were filled with complete medium with 10% FBS. After incubated for 24 h, the cells were fixed with methyl alcohol, followed by Giemsa staining. Transmembraned cells were dyed with 4′6-diamidino-2-phenylindole and counted under a fluorescence microscope (MOTIC, AE30/31). Each experiment was repeated for at least three times.

### Flow cytometry assay

Flow cytometric analysis was performed using a FACS Gallios (BD Biosciences, San Jose, CA, USA). AnnexinV-FITC and AnnexinV-APC staining kits were purchased from MultiSciences (#70-AP101-100) and were used to detect cell apoptosis. Each experiment was repeated for at least three times.

### Statistical analysis

Graphpad Prism application (Version 8.0.2.263) was used to analyze numerical data. For cellular experiments, each one was repeated for at least three times. At least 6 animals were in control and each treatment group. All data were expressed as mean ± SD. Statistical significance was determined by one-way analysis of variance (ANOVA) followed by Dunnett’s test. All data met normal distribution. *p* < 0.05 was considered statistically significant.

## Data Availability

All data generated or analyzed during this study are included in this published article.
